# South African parents’ views on oral, signing, and bilingual communication for Deaf or hard-of-hearing children

**DOI:** 10.4102/ajod.v13i0.1511

**Published:** 2024-12-20

**Authors:** Katijah Khoza-Shangase, Jasmine Bent

**Affiliations:** 1 Department of Audiology, Faculty of Human and Community Development, University of the Witwatersrand, Braamfontein, South Africa

**Keywords:** Deaf and Hard-of-Hearing (DHH) children, communication modes, South African context, parental decision-making, oral communication, sign language, bilingual communication, cultural influences, communication outcomes

## Abstract

**Background:**

Parents of Deaf or hard-of-hearing (DHH) children are faced with a plethora of overwhelming decisions concerning their children, particularly during the early stages of development. Among these decisions are those concerning assistive devices and the modes of communication for their child.

**Objectives:**

The aim of this study was to explore the perceptions of parents of DHH children towards the various modes of communication for their children within the South African context.

**Method:**

The study adopted a Q-methodology research design. Participants rated statements according to what they least and most agree with and then answered follow-up questions concerning the statements. Participants were also invited to participate in a live, one-on-one, semi-structured interview with the researcher. Data were analysed through both qualitative and quantitative statistics. Thematic analysis was adopted to analyse the qualitative data, while factor analysis through Ken-Q analysis was used for quantitative data.

**Results:**

Although 66% of participants thought that sign language allows DHH children to communicate more freely, 88% agreed that a DHH child should always learn to speak if they can. In terms of decision-making, 88% reported the issue of stigma or marginalisation and 88% cited the lack of Deaf schools as barriers in their decision-making.

**Conclusion:**

The study’s findings provide valuable insights into the complex interplay of factors influencing communication mode decisions for DHH children in South Africa.

**Contribution:**

These insights are crucial for developing inclusive and effective communication strategies that consider individual needs, societal norms and access to support services.

## Introduction

Deafness and other chronic hearing impairments continue to be among the most prevalent congenital conditions globally (Baron et al. 2019; Korver et al. 2017). To be Deaf or hard-of-hearing (DHH) certainly does not mean to be devoid of communication, there are varying modes of communication one could utilise, such as oral communication (spoken language), manual communication (sign languages such as South African Sign Language) and visual communication methods (gestures or visual aids). In addition, some parents may opt for a bilingual approach, integrating both spoken and sign languages to enhance communication flexibility and inclusivity (Casoojee & Khoza-Shangase 2022; Takala, Viljamaa & Fredäng 2018). Some of these modes of communication are associated with certain amplification devices that facilitate that mode of communication, such as cochlear implants (CIs).

Internationally, ever since the emergence of CIs in the late 1900s, numerous parents with DHH children who wish for their child to develop spoken language have opted for CIs as they believe that this amplification device will be more successful than the alternative of hearing aids (Bradfield 2021). While the number of CI surgeries continue to increase, CI services are still underused worldwide, with only a fraction of those qualifying for CI surgery actually undergoing the procedure, particularly in low-middle income contexts such as South Africa (Bhamjee et al. 2022; Casoojee & Khoza-Shangase 2022). This reality is because of a number of factors, the most common being patient or parent choice, financial constraints and patient age at diagnosis (Bhamjee et al. 2022; Casoojee & Khoza-Shangase 2022). While there have been measures taken to advance the South African public healthcare sector, there are still considerable limitations when it comes to financial resources and the provision of audiological services from prevention to rehabilitation (Casoojee & Khoza-Shangase 2022).

Results across studies have been inconsistent; however, children with no comorbidities who undergo CI surgery at a young age appear to have increased chances of successfully developing spoken language (Sharma et al. 2020; Baron et al. 2019). Be that as it may, there has been considerable pushback against CIs, particularly within the Deaf community, with some members likening the CI procedure to a form of genocide against the Deaf community (Bradfield 2021; Lee 2016). It is believed by some that sign language should be the preferred mode of communication for children who are DHH and that encouraging the use of spoken language instead separates them from their culture for the sake of mainstreaming into the hearing world (Bradfield 2021). This is especially persuasive, considering that the Deaf community are already a cultural minority, and thus the significance of sign language in preserving Deaf culture (Mauldin & Fannon 2021). Even still, this idea of mainstreaming into the hearing world is not considered concerning by all, with some suggesting that providing the child with speech and hearing will be of the most benefit to the child in a world that overwhelmingly favours those who are hearing and speaking (Bradfield 2021). This study’s focus is thus considered contentious for these reasons as a consensus is yet to be reached regarding the ethical implications of implanting or not implanting a CI and determining which mode(s) of communication children born with DHH should use (Bradfield 2021; Putnam et al. 2022). Studies have been conducted that examine this controversy from varying perspectives and with varying outcomes; however, there is a dearth of research, particularly from the African context, focusing on parents’ perceptions towards the various modes of communication for DHH children.

Parents’ perceptions play a crucial role in determining the communication routes chosen for DHH children. In South Africa, where approximately four to six in every 1000 babies are born with a hearing impairment, the decision-making process regarding communication modes for these children is complex and multifaceted (Davids, Roman & Schenck 2021; Joubert & Botha 2019). This decision about the communication mode choices is influenced by various factors such as parental beliefs, cultural considerations, access to resources such as CIs and the impact on the child’s social and linguistic development (Casoojee, Kanji & Khoza-Shangase 2021; Nichol 2020).

Parents play a crucial role in the decision-making process regarding communication modes for their DHH children, with their choices deeply influenced by their backgrounds, beliefs and personal experiences. Cultural and linguistic heritage often shapes parents’ preferences, with some prioritising spoken language to align with societal norms and perceived opportunities, while others may value sign language as a means of preserving Deaf culture and identity (Casoojee et al. 2021; McMenamin 2019). Religious beliefs, particularly those related to disability, can also influence decisions, with some parents viewing hearing loss as something to ‘overcome’ through oral methods (Kemmery 2014). Additionally, parents’ prior experiences with disability, whether through personal encounters or community interactions, can lead to varying degrees of openness to different communication methods.

The perceptions and choices of parents regarding these communication routes play a pivotal role in shaping the linguistic and social development of DHH children. Understanding the contextually relevant factors that influence these parental perceptions is crucial for designing effective interventions and support systems, including policies.

One of the primary influencing factors is personal judgement (Nichol 2020). Parents often weigh the benefits and challenges of each communication mode based on their understanding of their child’s needs, abilities and preferences. This includes considerations such as the child’s comfort with different communication methods, their proficiency in spoken language versus sign language and their overall communication goals (Hall & De Anda 2021; Nichol 2020). Cultural connections also play a significant role in parents’ decisions regarding communication modes for DHH children. Cultural identity and community affiliation can impact whether parents choose a communication mode that aligns with their cultural background and values. For example, parents who strongly identify with Deaf culture may prioritise sign language as the primary mode of communication for their child, emphasising cultural continuity and community connection (Bradfield 2021; Mauldin & Fannon 2021; Nichol 2020). Furthermore, healthcare professional advice and recommendations are essential influencers in the decision-making process. Parents often seek guidance from audiologists, speech-language pathologists and other healthcare professionals who specialise in working with DHH children. These professionals provide valuable insights into the potential benefits, challenges and outcomes associated with different communication modes, helping parents make informed decisions (Decker, Vallotton & Johnson 2012; Nichol 2020).

Understanding these influencing factors is crucial for developing effective communication strategies for DHH children. It requires a holistic approach that considers the child’s individual needs, cultural background and professional guidance to ensure optimal communication outcomes and support their overall development and well-being. This article, therefore, aims to explore South African parents’ attitudes and perceptions towards the oral, signing and bilingual routes of communication for their DHH children. By delving into the motivations, beliefs and challenges faced by parents in making these decisions, this study seeks to contribute to a deeper understanding of the factors driving communication mode choices in this population. Ultimately, the insights gained from this research can inform policies and practices aimed at enhancing the communication outcomes and overall well-being of DHH children in South Africa.

The gap in research from the African context could emphasise the unique cultural, social and economic factors in South Africa that significantly influence parental decisions regarding communication methods for DHH children. In South Africa, the linguistic diversity, with 11 official languages and Sign Language, creates challenges in accessing resources and support in a child’s home language, particularly for sign language (Davids et al. 2021; Maluleke, Khoza-Shangase & Kanji 2021; Shezi & Joseph 2021). Additionally, the historical marginalisation of sign language and the limited availability of schools for the DHH further constrain parental choices (Holness 2016; Senne 2016). Economic disparities also play a critical role, as access to cochlear implants and hearing aids is often limited to those with financial means, leaving many parents to rely on less expensive, sometimes less effective, communication methods that they can only access from the public healthcare system that is overburdened. Socioeconomic status further impacts these decisions, as access to resources such as cochlear implants, specialised education and speech therapy can be limited, pushing parents to make choices based on what is available rather than what might be most effective. Ultimately, these diverse factors contribute to a complex and deeply personal decision-making process, where parents must balance their aspirations for their child’s future with practical realities and deeply held values. Furthermore, societal attitudes towards disability, influenced by cultural beliefs and stigmas, can pressure parents to prioritise spoken language acquisition over other communication methods (Mkabile & Swartz 2020). These factors collectively shape the complex landscape in which South African parents make decisions for their DHH children, highlighting the need for culturally and contextually sensitive research and support.

The theoretical framework underpinning this study is grounded in the models of communication for DHH children, which encompass oral, signing and bilingual approaches. These models are informed by linguistic, cognitive and sociocultural theories that emphasise the importance of early and effective communication in the cognitive and social development of DHH children (Easterbrooks 2020). The oral approach is rooted in auditory-verbal therapy and speech-language pathology, focusing on the development of spoken language through residual hearing and speechreading. In contrast, the signing approach is based on visual-manual communication, primarily using sign language as a natural language for DHH individuals, promoting linguistic accessibility and cultural identity within the Deaf community. The bilingual approach integrates both spoken and sign languages, recognising the cognitive benefits of bilingualism and promoting a dual-linguistic pathway for DHH children (Easterbrooks 2020; Hatrak 2022; Pizzo 2016). These models serve as the foundation for exploring the parental decision-making process in the South African context, where cultural, social and economic factors play a crucial role in shaping communication choices for DHH children.

## Methods

### Aim

The primary goal of this study was to delve into South African parents’ perceptions regarding the oral, signing and bilingual routes of communication for their children who are DHH.

### Objectives

The specific objectives were twofold:

1. To delve into parents’ perceptions regarding the oral, signing and bilingual routes of communication.
2. To investigate the factors and motivators that influence parents’ decisions regarding the modes of communication their child will adopt.

### Research design

This study employed Q-methodology and qualitative analysis of semi-structured interviews, both of which were chosen to capture the complexities of parents’ perspectives on communication modes for DHH children. These methods complement each other, allowing for a mixed-methods approach that provides both breadth and depth in understanding parental decision-making processes. Q-methodology was selected because it is particularly well suited for exploring subjective viewpoints and identifying patterns of shared beliefs among participants (Akhtar-Danesh, Baumann & Cordingley 2008; Gao & Soranzo 2020; Klein 2022). This method helps to organise complex attitudes into manageable clusters, making it ideal for a study that seeks to understand diverse parental perspectives on communication options. By asking participants to rank statements related to communication choices, the Q-methodology enabled the grouping of like-minded individuals and revealed trends in their preferences. This clustering, based on Pearson’s correlation coefficient and Horst’s centroid method, allowed us to identify distinct ‘factors’ or clusters of parents with similar views (Klein 2022). Q-methodology provided structure to the data by offering a quantitative way to capture the subjective experiences of parents, thus balancing the rich, qualitative insights from interviews with statistically supported clusters of attitudes. The qualitative analysis was conducted following semi-structured interviews to delve deeper into the personal, cultural and emotional factors that influence parents’ decisions. Semi-structured interviews were chosen for their flexibility, enabling participants to express their views while the researcher maintained the ability to probe specific areas of interest. This method is appropriate for exploratory research where participants’ experiences and reflections are key to understanding complex decision-making processes.

The instrument used in this study, distributed using the Survey Planet platform, comprised a list of 27 statements, in English, that participants rated according to what they most and least strongly agree with (Damio 2016). This exercise took approximately 10 min – 20 min to complete. The statements represented the broad but generalised range of viewpoints one would have on the topic (Klein 2022). For example, in this case, statements ranged from: ‘It is not necessary for a Deaf or hard-of-hearing child to learn how to speak’. to ‘If a Deaf or hard-of-hearing child can learn to speak, they always should’. Following this rating, participants were asked to answer a few open-ended questions concerning the topic. Finally, they were invited to participate in an additional, semi-structured interview to discuss the topic and elaborate on their responses further. Questions such as: ‘What were the key reasons behind your agreement or disagreement with the statements in the survey?’; ‘Do you think your viewpoint is common or do you feel that you are in a minority who feel this way?’; ‘Would you say that your agreement or disagreement with the statements was influenced by outside factors (e.g., conversations with medical practitioners, conversations with other parents or loved ones, online resources, the media, etc.)? If yes, what were the main external factors?’, formed the interview guide. These interviews took place either over the phone or in-person, depending on what was more convenient for the participant, and lasted for approximately 15 min – 30 min. The interview was audio recorded and then transcribed for analysis. This approach allowed for a blend of qualitative depth and quantitative analysis, particularly beneficial for subjective topics such as perceptions of communication routes (Damio 2016; Gao & Soranzo 2020; Klein 2022).

### Research site

The initial research site was The Children’s Communication Centre in Houghton, Johannesburg, which focuses on teaching DHH children oral and/or auditory skills in English. However, because of a low response rate from targeting only one institution, a research poster was designed and shared online via social media (Facebook, X [formerly Twitter], WhatsApp) to enhance participation.

### Participants

The study employed non-probability convenience sampling and voluntary response sampling strategies. Nine participants met the survey criteria, with an additional five participants interviewed for more detailed insights (Klein 2022; McCombes 2023). The selection criteria included parents or legal guardians of DHH children who were South African citizens or permanent residents. Minors and individuals unable to provide informed consent were excluded.

### Data collection procedures

The Q-set used in the study consisted of statements reflecting different viewpoints, sourced from relevant literature (Bradfield 2021; Chang 2017; Crowe et al. 2014; Gale 2010; Mauldin 2012; Nichols 2018). Data collection procedures included obtaining ethical clearance (Protocol Number: STA_2023_15), conducting a pilot study for refinement and then administering online surveys, with additional interviews for deeper insights. Ethical principles outlined in The Belmont Report and HPCSA General Ethical Guidelines were strictly adhered to throughout the study.

### Data analysis

Thematic analysis was employed for qualitative data, while factor analysis, specifically Ken-Q Analysis, was used for quantitative data to group participants based on their responses (Du Plessis 2019; Klein 2022; Nowell et al. 2017; Vaismoradi, Turunen & Bondas 2013). The qualitative data in this study were analysed using a detailed and systematic approach to thematic analysis (Nowell et al. 2017). An inductive thematic analysis was adopted following Braun and Clarke’s (2006) approach to analyse the semi-structured interview data. This method was specifically selected because of its flexibility and its capacity to identify, analyse and report patterns across the data set in rich detail. An inductive approach meant that the themes identified were strongly linked to the data themselves, without being influenced by pre-existing theories or frameworks. This was crucial for this study, as it aimed to explore South African parents’ perceptions and decisions regarding communication modes for their DHH children, a topic where cultural, social and personal nuances might emerge organically.

Braun and Clarke’s six-step process was followed: (1) transcription of the interview data and familiarisation with the data through repeated reading of interview transcripts, (2) data segmentation into meaningful units or codes, which are portions of the text that convey distinct ideas or concepts, that is, generating initial codes based on meaningful data segments, (3) codes grouped into broader themes that captured the essence of the participants’ responses, searching for themes among these codes, (4) reviewing the themes to ensure they accurately captured the data, (5) defining and naming themes to fully represent the essence of each and (6) producing the final report. In line with the approach suggested by Vaismoradi et al. (2013), these themes were not predetermined but rather emerged inductively from the data. This allowed the researchers to capture the complexity and depth of the participants’ experiences and perceptions. This method was deemed appropriate given the complexity of the subject matter, where multiple perspectives and experiences needed to be understood in depth. Additionally, this approach allowed for the capturing of both commonalities and differences in the participants’ narratives, making it ideal for the exploratory nature of this research. By utilising Braun and Clarke’s thematic analysis, the study ensured that the themes were data driven, providing a robust representation of parents’ lived experiences and decision-making processes without imposing preconceived categories. The final themes were then organised and described in detail, providing a rich and nuanced understanding of the participants’ perspectives on the communication choices for their DHH children. This methodical approach to thematic analysis ensured that the findings were grounded in the data, providing a trustworthy and credible interpretation of the participants’ experiences. This method, grounded in qualitative rigor, offers transparency in the analysis and contributes to the overall trustworthiness of the findings.

The quantitative data were analysed using factor analysis, which allowed for the identification of two distinct groups or ‘clusters’ of participants based on their similar response patterns. The participants were divided into clusters according to the correlation matrix. This clustering process was facilitated through Ken-Q analysis, which groups participants according to Pearson’s correlation coefficient and Horst’s centroid method of analysis (Du Plessis 2019; Klein 2022). The correlation matrix generated through this analysis provided a visual representation of how closely each participant’s responses correlated with those of others. Participants who exhibited similar views were grouped into clusters, effectively highlighting patterns of agreement and disagreement within the sample. For instance, participants within the same cluster shared similar attitudes towards specific statements, while those in different clusters had diverging opinions.

By analysing these clusters, the study was able to compare the statements that each group of participants agreed or disagreed with, shedding light on underlying trends and commonalities within the data. This approach also allowed for the identification of the most populated clusters and highlighted any statements that received unanimous agreement or disagreement across clusters, providing deeper insights into the participants’ collective attitudes and perceptions (Akhtar-Danesh et al. 2008; Gale 2010).

### Trustworthiness and rigour

The study ensured confirmability, dependability and rigour through documentation, triangulation of data sources and participant expansion on survey responses (Klein 2022; Nielsen et al. 2020; Nowell et al. 2017).

### Reliability and validity

Content validity was established through a pilot study and face validity was maintained by using accessible language in the survey. Q-sorting validity was upheld by employing user-friendly software for data analysis (Akhtar-Danesh et al. 2008; Klein 2022).

### Ethical considerations

This study was approved by the Human Research ethics committee of the University of the Witwatersrand (protocol number: STA_2023_15). The World Medical Association (WMA) Declaration of Helsinki (2013) guided the ethical considerations for this study.

## Results

### Participants’ profile

In line with the inclusion criteria, all participants were parents or legal guardians of DHH children. A large majority (78%) of the participants’ children did not attend schools for the DHH, with only 22% enrolled in them. Of the total sample, 22% reported that their children do not use any hearing assistive devices, 33% reporting occasional use and only 45% reporting their children using these always. As far as communication mode used by the DHH children was concerned, a variety of modes of communication were reported, with speech and gestures being used by 35% of the participants, respectively, and sign language by only 12% of the sample, with the rest (18%) using a combination.

The participants were divided into clusters according to the correlation matrix depicted in Table 1. Participants 2, 3 and 4 form one cluster, while P1, 5, 6, 7, 8 and 9 formed the other. The differing attitudes of the two clusters will be referred to when discussing the results of the study. The closer the number is to 100, the closer those respondents’ responses correlated with each other.

**TABLE 1:** Correlation matrix of survey results (*N* = 9).

Respondent	P1	P2	P3	P4	P5	P6	P7	P8	P9
P1	100	−23	−20	−20	21	41	16	25	38
P2	−23	100	56	5	−42	−25	−16	−39	−28
P3	−20	56	100	25	−32	1	−15	−3	−8
P4	−20	5	25	100	13	−5	−34	−9	5
P5	21	−42	−32	13	100	18	9	22	−19
P6	41	−25	1	−5	18	100	19	12	4
P7	16	−16	−15	−34	9	19	100	37	23
P8	25	−39	−3	−9	22	12	37	100	35
P9	38	−28	−8	5	−19	4	23	35	100

P, participant.

### Exploring parents’ perceptions of the oral, signing and bilingual modes of communication

**FIGURE 1:**
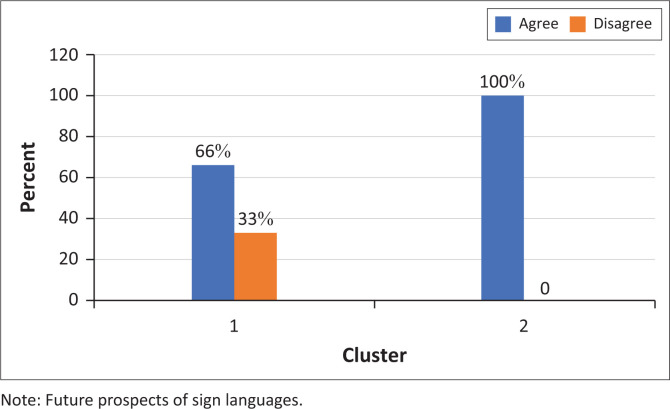
Participants’ responses to the statement ‘sign languages will be used more and more in the future’ (*N* = 9).

**FIGURE 2:**
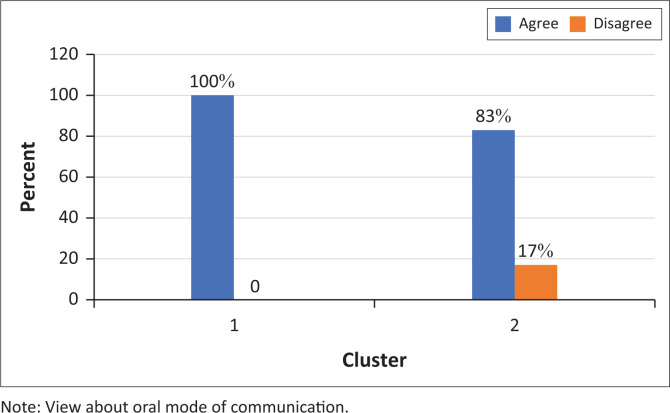
Participants’ responses to the statement ‘If a Deaf child can learn to speak, they always should’ (*N* = 9).

Although most participants (88%) agreed that ‘Sign languages will be used more and more in the future’ all but one participant felt that a Deaf child should always learn to speak whether they are capable (Figure 1 and Figure 2).

**FIGURE 3:**
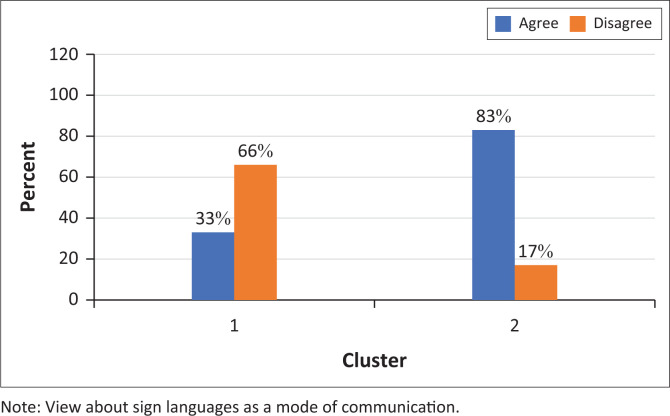
Participants’ responses to the statement ‘Sign languages allow a Deaf child to communicate more freely’ (*N* = 9).

**FIGURE 4:**
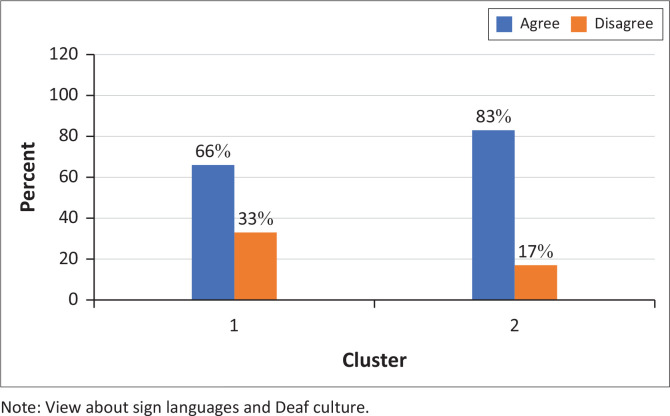
Participants’ responses to the statement ‘Sign languages help to preserve Deaf culture’. Most participants agreed with the statement (*N* = 9).

Cluster 2 participants agreed that ‘Sign languages allow a Deaf child to communicate more freely’ (83%) and were more likely to agree that ‘Sign languages help to preserve Deaf culture’ (83%) (Figure 3 and Figure 4).

**FIGURE 5:**
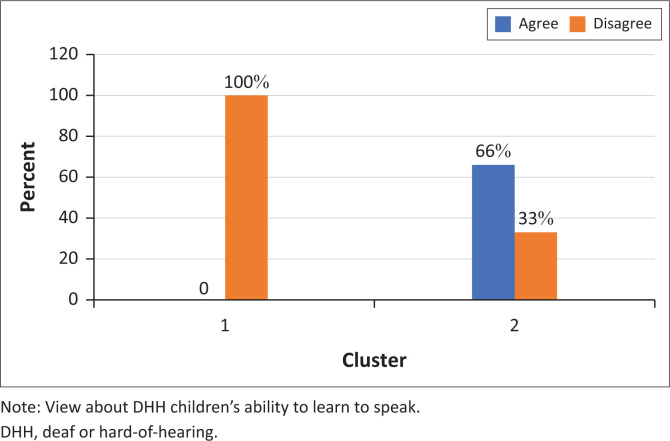
Participants’ responses to the statement ‘Deaf or hard-of-hearing children cannot learn to speak as well as hearing children’ (*N* = 9).

**FIGURE 6:**
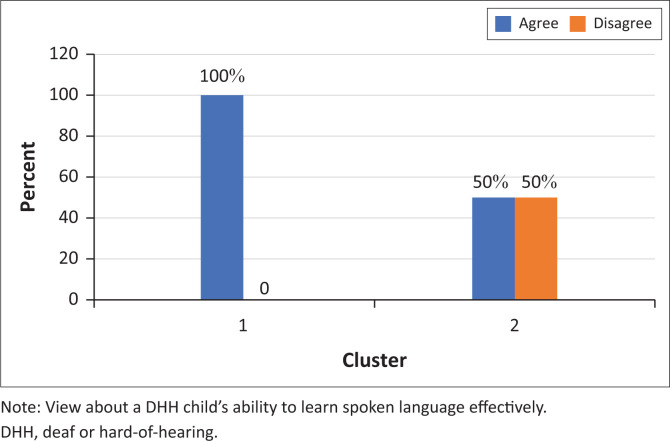
Participants’ responses to the statement ‘Deaf or hard-of-hearing children can learn spoken language effectively’ (*N* = 9).

**FIGURE 7:**
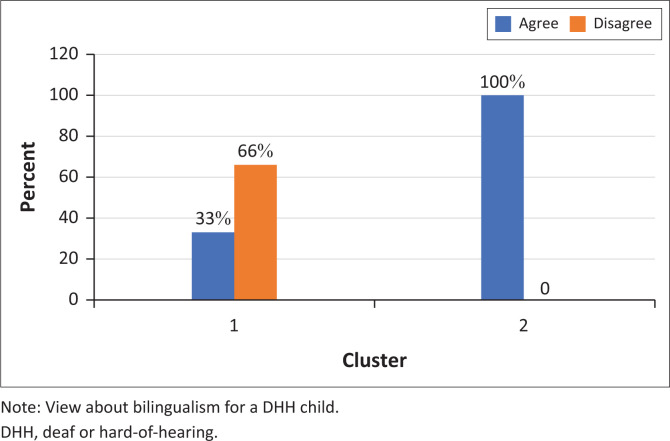
Participants’ responses to the statement ‘A Deaf or hard-of-hearing child should learn a sign and spoken language at the same time’ (*N* = 9).

Participants in cluster 1 were all of the belief that DHH children can learn to speak as well as their hearing peers (Figure 5 and Figure 6). They also did not think that it is effective for a DHH child to learn a sign and spoken language at the same time (Figure 7). As opposed to cluster 2 participants who were less likely to think that DHH children can learn how to speak as well as their hearing peers (only 33%).

**FIGURE 8:**
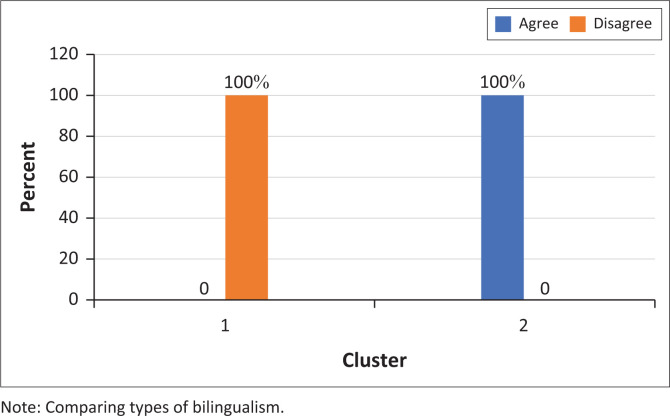
Participants’ responses to the statement ‘Being able to use both a sign and spoken language can be compared to speaking both English and Zulu’ (*N* = 9).

A notable distinction was in the participants’ perception of sign languages as valid languages. One of the most distinctive statements in the survey was ‘Being able to use both a sign and spoken language can be compared to speaking both English and Zulu’. All cluster 1 participants disagreed, while all cluster 2 participants agreed (Figure 8). In addition, one of the cluster 1 participants remarked:

‘I don’t think sign language is a language truly. When you talk of language you should be able to speak and communicate verbally and not just with signs.’ (Participant 2, Parent, Female 38)

### Investigating the influencing factors or motivators behind parents’ decisions concerning the modes of communication their child will use

**FIGURE 9:**
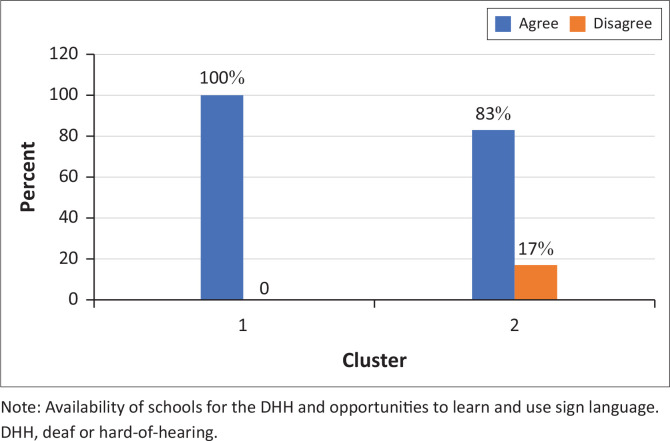
Participants’ responses to the statement ‘There are not enough Deaf schools in South Africa where Deaf or hard-of-hearing children can learn and use sign language’ (*N* = 9).

When it came to the influencing factors behind the decision of what mode of communication their child should use, two major themes emerged: (1) *marginalisation* and *ostracism* and (2) *nature of disability*. The most prominent theme, which had six sub-themes (parental advocacy and empowerment, resource availability and accessibility, cultural and social integration, identity and belonging, impact on family dynamics and long-term outcomes and future prospects), was that of *marginalisation* and *ostracism.* Many participants did not feel that their child would have the necessary accommodations made for them if they were to opt for the signing route. This corroborates with the survey results that reflect that all but one participant (88%) agreed with the statement ‘There are not enough Deaf schools in South Africa where DHH children can learn and use sign language’ (Figure 9).

The following quotations under the six sub-themes highlighting the issue of ‘marginalisation and ostracism’:

One sub-theme was ‘parental advocacy and empowerment’, where parents expressed a strong sense of responsibility in advocating for their child’s needs and accessing appropriate resources. This was closely linked to the sub-theme of ‘resource availability and accessibility’, as many parents described how the scarcity of resources – particularly for sign language – affected their decisions. Parents often had to weigh the availability of professional support against their desired mode of communication.

### Parental advocacy and empowerment

‘As a mom, I felt it was my job to make sure that my child had access to every possible opportunity, whether that meant fighting for resources at school or learning sign language as a family.’ (Participant 1, Parent, Female 41)

‘We had to speak for our child … become his voice in a system that doesn’t always understand what he needs.’ (Participant 9, Parent, Male 40)

‘Generally, I think if they can afford to learn to speak English it would be good for them because most of the people that will deal with them, it’s not all of them that will necessarily understand their own language.’ (Participant 3, Guardian, Female 41)

### Resource availability and accessibility

‘We chose the oral mode mainly because the resources for sign language are so limited in our area … there are no schools for the Deaf.’ (Participant 3, Guardian, Female 41)

‘The lack of trained professionals who can support sign language was a major barrier for us.’ (Participant 2, Parent, Female 38)

‘Accessibility and availability of schools and trained people is a big deal. I mean, there’s not many schools that are available even for kids that have these different abilities never mind within a normal, mainstream school.’ (Participant 6, Parent, Female 39)

Another sub-theme was ‘cultural and social integration’, which highlighted the pressures parents felt from their communities to choose spoken language as the primary mode of communication. This was in contrast to the sub-theme of ‘identity and belonging’, where parents discussed the importance of connecting their children to the Deaf community and fostering a strong sense of Deaf identity through the use of sign language.

### Cultural and social integration

‘I think it’s a good thing to learn. I just think sign language is not even normalised in South Africa in and of itself. Even with parents and their children who are hard-of-hearing, it’s not a normalised habit. Even if we train teachers and educators, there’s not a continued education for children in sign language but I think it is a great thing to develop.’ (Participant 6, Parent, Female 39)

‘In our community, spoken language is seen as essential for success, so we felt pressured to choose oral communication.’ (Participant 1, Parent, Female 41)

‘We wanted our child to be able to communicate with their hearing family members and friends, which influenced our decision towards oral communication.’ (Participant 8, Parent, Female 42)

### Identity and belonging

‘But for Deaf children who are not multi-disabled I think it is very important to also learn spoken language. Deaf people using only sign language are very cut off from society at large and cannot communicate well doing shopping et cetera, and cannot enter more or less all occupations. So they are more cut off from society.’ (Participant 1, Parent, Female 41)

‘We didn’t want our child to feel isolated from the Deaf community, so we made sure he learned sign language.’ (Participant 3, Guardian, Female 41)

‘It’s important for us that our child embraces their Deaf identity, and sign language is a big part of that.’ (Participant 9, Parent, Male 40)

The theme of ‘impact on family dynamics’ revealed how the decision to adopt a particular communication mode was not only about the child but also about what worked for the entire family.

### Impact on family dynamics

‘Learning sign language together as a family brought us closer and helped us better understand each other.’ (Participant 2, Parent, Female 38)

‘The decision we made had to work for the entire family, not just for our child.’ (Participant 4, Guardian, Female 38)

Finally, the theme of ‘long-term outcomes and future prospects’ reflected parents’ considerations of how their communication choices would affect their children’s opportunities in education, employment and broader social integration.

### Long-term outcomes and future prospects

‘We thought about which mode of communication would offer the best future opportunities for our child, you know … whether in education or employment.’ (Participant 6, Parent, Female 39)

‘We hope that by choosing oral communication, our child will have an easier time being part of the society as a whole … and our extended family.’ (Participant 1, Parent, Female 41)

The second theme was ‘nature of disability’. One of the participants whose child exclusively uses sign language, for example, cited the nature of his child’s disability as the reason behind choosing this mode of communication. Perceptions of deafness as either a disability or difference, as well as medical versus social models of disability, emerged as sub-themes under this theme.

### Perceptions of deafness as a disability versus difference

‘Some people see our child’s deafness as a limitation, but we view it as just another way of experiencing the world.’ (Participant 3, Guardian, Female 41)

‘We don’t see our child as disabled; we see him as differently-abled, and that influenced our decision to embrace sign language.’ (Participant 6, Parent, Female 39)

‘Being multi-disabled, [*my child*] could only learn to speak a few words, but took to sign language immediately, so that is her primary language.’ (Participant 1, Parent, Female 41)

### Medical versus social models of disability

‘The doctors kept pushing us towards cochlear implants, making it seem like deafness is something broken that needed to be fixed.’ (Participant 4, Guardian, Female 38)

‘We felt that the focus should be on changing the environment …everything around us … to our child’s needs, rather than trying to ‘normalise’ their hearing.’ (Participant 8, Parent, Female 42)

## Discussion

The study’s participants were parents or legal guardians of DHH children in South Africa. The demographic data revealed several key insights into their experiences and perspectives regarding communication modes and educational settings for their children. The fact that a large majority (78%) of the participants’ children did not attend schools for the DHH suggests a significant proportion of DHH children are integrated into mainstream educational settings rather than specialised schools for the DHH in this sample. This might be for various reasons, including the lack of specialised schools for the DHH in the country. This limitation in access seems also to be possibly evident in the use of hearing assistive devices, where only 78% of participants’ children used hearing assistive devices, primarily hearing aids, although all would have benefited from them based on their hearing function. Furthermore, the fact that only 45% used them consistently may also highlight potential challenges or limitations in access to and utilisation of these devices.

A diversity of communication modes was found among the participants’ children. While speech and gestures were common (35% each), sign language was less prevalent (12%), with the combination of modes used by18% of the sample. This distribution reflects the varied approaches adopted by families in facilitating communication for their DHH children. This is similar to Nichol’s (2020) study where a minority of the participants’ children used sign language. Notably, all participants in this study reported that they themselves were hearing and communicated using speech, in line with the statistic that the vast majority of parents with DHH children are hearing and speaking (Bradfield 2021). Similar patterns were evident in Chang’s (2017) study where most parents were hearing (76%) and thus opted for CI surgery for their child. The profile of this study is thus skewed towards parents whose children use the oral route of communication and use hearing assistive devices.

The study delved into parents’ perceptions regarding oral, signing and bilingual communication modes for DHH children. Two distinct clusters emerged based on the correlation matrix, reflecting differing attitudes and beliefs among participants. Firstly, as far as sign language usage was concerned, Cluster 2 participants were more supportive of sign language, perceiving it as facilitating freer communication and preserving Deaf culture. In contrast, Cluster 1 participants emphasised the importance of learning spoken language, with some scepticism about the efficacy of sign language. Secondly, as far as spoken language acquisition was concerned, while most participants agreed that sign languages would gain prominence in the future, there was a strong consensus that DHH children should learn to speak if capable. Cluster 1 participants believed DHH children can learn to speak effectively, whereas Cluster 2 participants were less optimistic about this ability. Current findings are congruent with available evidence indicating that the oral route is more commonly chosen among parents of DHH children (Bradfield 2021; Nichol 2020).

The issue of the preservation of Deaf culture is acknowledged by parents in this study as well as previous literature (Bradfield 2021; Chang 2017). Most participants in this study, those constituting Cluster 2, believed that sign language helps to preserve Deaf culture but also that people with CIs are still a part of the Deaf community. Parents in Chang’s (2017) study mostly felt that their children were shunned or not accepted by the Deaf community once they had CIs. Overall, parents across all contexts generally appear to be of the belief that spoken language opens more opportunities for their child in a hearing-centred world. These findings make it evident that parents within the South African context are concerned about the barriers associated with using sign language and this often motivates them to choose the oral mode of communication. There is also an issue surrounding the characterisation of sign language as mere gestures rather than a valid language, as expressed by a couple of participants in this study. Overall, there may be a need to raise awareness about the value and use of sign language in South Africa to prevent the marginalisation of those who sign.

Regarding the influencing factors or motivators behind communication mode decisions, the study uncovered two significant themes related to the factors influencing parents’ decisions for their DHH children. ‘Marginalisation and ostracism’ is one of the themes identified where a prevalent concern among participants was the fear of marginalisation and a lack of accommodation if their child pursued sign language. This fear stemmed from perceptions of limited access to schools for the DHH and societal norms favouring spoken language. Sub-themes with their accompanying quotes from participants highlighted these concerns, reflecting broader challenges faced by DHH individuals in accessing education and social inclusion within the South African context. While parents in Nichol’s (2020) study were vocal about the ostracism endured by people who sign, there were differences with regard to their primary concerns compared to this study. Participants in that study were primarily concerned with the child’s ability to communicate with their home community and communicate like the rest of the family. Those participants were also less concerned about the child’s ability to lead a ‘normal’ life and fit in with their peers. On the other hand, parents in this study reported concerns with regard to the prejudice the child would face from the broader society and not just their home community. The issues of being cut off from society and discriminated against were also brought up more frequently by participants in this study.

The six sub-themes under this theme reveal the complex interplay of factors that influence parental decisions regarding communication modes for DHH children. Parental advocacy and empowerment emerge as crucial, with parents taking active roles to ensure their children’s access to opportunities, whether by advocating within the educational system or learning sign language themselves. However, the availability and accessibility of resources significantly shape these decisions, as some parents opt for oral communication because of the scarcity of support for sign language in the South African context. Cultural and social integration pressures also weigh heavily, with parents choosing oral communication to align with community expectations and facilitate their child’s interaction with hearing family members. At the same time, concerns about identity and belonging lead other parents to prioritise sign language, recognising its importance in fostering a connection with the Deaf community and embracing a Deaf identity. The impact on family dynamics is also notable, with shared language learning fostering closer relationships, while decisions are made with consideration for the entire family’s needs. Finally, long-term outcomes and future prospects guide parental choices, with a focus on which communication mode will best support their child’s educational and employment opportunities and their integration into broader society. These sub-themes underscore the multifaceted nature of parental decision-making in the context of DHH children’s communication options.

‘Nature of Disability’ is the second major theme that emerged. Some participants cited the nature of their child’s disability as a decisive factor in choosing a communication mode. For instance, one participant mentioned their child’s multidisability as influencing the preference for sign language. This finding underscores the individualised nature of communication decisions based on a child’s specific needs and abilities. The two sub-themes under this theme highlight the contrasting perceptions of deafness as either a disability or a difference, which significantly influence parental decisions regarding communication methods for their DHH children. Some parents reject the notion of deafness as a limitation, instead viewing it as a unique way of experiencing the world, which leads them to embrace sign language and other communication methods that affirm their child’s identity. This perspective aligns with the social model of disability (Harvey 2008; Shakespeare 2006), where the focus is on adapting the environment to meet the needs of the child, rather than attempting to ‘fix’ the child through medical interventions like cochlear implants (Harvey 2008; Power 2005). In contrast, the medical model, often advocated by healthcare professionals, emphasises the correction of deafness through technology, framing it as a condition to be remedied (Kelly-Corless 2024). These differing viewpoints reflect a broader debate between medical and social approaches to disability, with significant implications for how parents choose to support their child’s communication and integration into society.

This study’s results are deeply intertwined with the socio-cultural and educational landscape of South Africa, where access to inclusive education and specialised services for DHH individuals can vary significantly. The challenges highlighted, such as limited schools for the DHH availability, societal attitudes towards sign language where discrimination is a key barrier against parents’ willingness to choose the signing mode of communication and concerns about integration and inclusion, resonate with broader issues within the South African context. The need for schools for the DHH and sign language education in the South African context is also highlighted.

This study sought to explore South African parents’ perceptions of the oral, signing and bilingual modes of communication for their DHH children. Through a detailed examination of participants’ responses, the study successfully achieved its objectives by identifying the prevailing attitudes, beliefs and factors influencing parental decisions regarding communication modes. The results revealed a significant inclination towards oral communication, with a strong preference for spoken language development, even among parents who acknowledged the importance of sign language. The clustering of participants based on their responses provided a nuanced understanding of the diverse perspectives, particularly highlighting the tension between cultural preservation through sign language and the perceived practical advantages of oral communication. The study also uncovered the critical role of marginalisation and accessibility issues in shaping these decisions, thereby providing a comprehensive overview of the complexities parents face in choosing the most suitable communication mode for their children. By addressing these objectives, the study has contributed valuable insights into the discourse on DHH education and parental choice in the South African context.

## Conclusion

In conclusion, while this study provides valuable insights into parental perceptions and decision-making regarding communication modes for DHH children in South Africa, it also highlights the need for continued research, policy advocacy and community engagement to create a more inclusive and supportive environment for DHH individuals and their families. The need to expand the exploration of communication methods beyond the primary focus on the dichotomy between cochlear implants and sign language provided in this study is acknowledged. To provide a more comprehensive understanding of the available options for parents of DHH children, future work will include a more detailed examination of bilingual approaches that integrate both spoken and sign languages. By incorporating this perspective, a holistic view that recognises the diversity of communication strategies will be offered, ultimately providing parents with a broader range of evidence-based options for their children’s communication development.

Current findings should be interpreted with the identified methodological limitations in mind. Firstly, the study’s sample size was relatively small, comprising nine participants, which may limit the generalisability of the findings to a larger population of parents or legal guardians of DHH children in South Africa. Secondly, the study primarily focused on participants who were already engaged with the Children’s Communication Centre or accessed through social media, potentially leading to a biased sample that may not fully represent the diversity of perspectives within the broader population. Thirdly, as with any self-reporting study, there may be inherent biases in how participants perceive and report their experiences and beliefs, which could influence the validity of the results. To ensure academic rigour and transparency, the study employed several strategies to address and mitigate these limitations. Initially, acknowledging the small sample size as a potential limitation, the study utilised in-depth qualitative methods to extract rich, detailed data from each participant. This approach allowed for a more nuanced understanding of the participants’ experiences and perceptions, compensating for the limited number of respondents. Additionally, to counter the limitation of potential bias in participant selection, the study employed purposive sampling. This method ensured that participants were selected based on specific criteria relevant to the research question, thereby increasing the relevance and depth of the data collected. The use of thematic analysis in the qualitative component further strengthened the study’s rigour by systematically identifying and analysing patterns and themes across the data. This method, grounded in established frameworks (Nowell et al. 2017), ensured that the analysis was both thorough and consistent. Furthermore, the study’s reliance on triangulation – integrating quantitative factor analysis with qualitative thematic analysis – helped to cross-validate the findings. This approach not only provided a more comprehensive understanding of the data but also enhanced the credibility and robustness of the results. To address potential researcher bias, reflexivity was maintained throughout the research process, with the researchers continuously reflecting on their assumptions and influences to ensure that the analysis remained objective and grounded in the data. Lastly, the study’s transparency was demonstrated through detailed documentation of the research process, including the data collection methods, analytical procedures and interpretation of results. By clearly outlining these steps, the study ensured that its findings could be scrutinised, replicated or built upon in future research, thus contributing to the field’s ongoing discourse.

Bearing the aforesaid in mind, the study raises some implications. Firstly, the findings underscore the need for educational policies and initiatives that promote inclusive education and provide adequate resources and support for DHH children in both mainstream and specialised educational settings. Secondly, there is a clear need for increased awareness and training among healthcare professionals, educators and the general public regarding the diverse communication needs of DHH children and the importance of accommodating these needs effectively. Thirdly, addressing societal attitudes and biases towards sign language and Deaf culture is essential for creating an inclusive and supportive environment that values linguistic diversity and promotes social inclusion for DHH individuals. Therefore, there are several recommendations that can be made. Conducting larger-scale studies with a more diverse participant pool can provide deeper insights into the complex factors influencing communication mode decisions for DHH children in South Africa. Additionally, there is a need to engage with Deaf communities, advocacy groups and relevant stakeholders to co-create communication strategies and policies that are sensitive to cultural diversity and promote inclusion. Furthermore, ongoing training and professional development opportunities for healthcare professionals, educators and support personnel to enhance their understanding of DHH communication needs and best practices are needed. Lastly, advocating for policy changes and initiatives that prioritise access to specialised services, inclusive education, and linguistic rights for DHH individuals, including the recognition and promotion of sign language as valid and valuable form of communication is recommended.
